# Curcumin-primed milk-derived extracellular vesicles remodel hair follicle microenvironment for the treatment of androgenetic alopecia

**DOI:** 10.1093/rb/rbaf051

**Published:** 2025-05-30

**Authors:** Chongchao Hou, Sihua Wang, Zihang Li, Qing Huang, Yang Jiang, Xin Zhou, Rongying Ou, Danyang Li, Yunsheng Xu

**Affiliations:** Department of Dermatovenereology, The Seventh Affiliated Hospital, Sun Yat-sen University, Shenzhen 518107, China; Research Center, The Seventh Affiliated Hospital, Sun Yat-sen University, Shenzhen 518107, China; Department of Dermatovenereology, The Seventh Affiliated Hospital, Sun Yat-sen University, Shenzhen 518107, China; Research Center, The Seventh Affiliated Hospital, Sun Yat-sen University, Shenzhen 518107, China; Department of Dermatovenereology, The Seventh Affiliated Hospital, Sun Yat-sen University, Shenzhen 518107, China; Research Center, The Seventh Affiliated Hospital, Sun Yat-sen University, Shenzhen 518107, China; Research Center, The Seventh Affiliated Hospital, Sun Yat-sen University, Shenzhen 518107, China; Department of Geriatrics, The Seventh Affiliated Hospital, Sun Yat-sen University, Shenzhen 518107, China; Research Center, The Seventh Affiliated Hospital, Sun Yat-sen University, Shenzhen 518107, China; Department of Interventional Oncology, The First Affiliated Hospital, Sun Yat-sen University, Guangzhou 510080, China; Department of Dermatovenereology, The Seventh Affiliated Hospital, Sun Yat-sen University, Shenzhen 518107, China; Research Center, The Seventh Affiliated Hospital, Sun Yat-sen University, Shenzhen 518107, China; Department of Gynaecology and Obstetrics, The First Affiliated Hospital, Wenzhou Medical University, Wenzhou 325000, China; Research Center, The Seventh Affiliated Hospital, Sun Yat-sen University, Shenzhen 518107, China; Department of Dermatovenereology, The Seventh Affiliated Hospital, Sun Yat-sen University, Shenzhen 518107, China; Research Center, The Seventh Affiliated Hospital, Sun Yat-sen University, Shenzhen 518107, China

**Keywords:** androgenetic alopecia, curcumin, milk-derived extracellular vesicles, hair follicle microenvironment, hair follicle cycle

## Abstract

Androgenetic alopecia (AGA) is a globally prevalent condition, with limited treatment options and significant adverse effects associated with existing therapies. The primary pathogenic mechanisms of AGA involve androgen-mediated regulatory pathways, molecular alterations affecting hair regeneration, and inflammation in the perifollicular microenvironment. In this study, we first investigated the topical application of testosterone with varied doses for AGA mouse model induction, in which the High-dose group exhibited the most robust model development and provided a more comprehensive set of criteria for successful AGA model establishment. Then, curcumin-primed milk-derived extracellular vesicles (Cur-mEVs) were fabricated for the therapy of AGA with the in-house developed mouse model described above. It was demonstrated that Cur-mEVs remodeled the hair follicle microenvironment, evidenced by the activation of the Wnt/β-catenin signaling pathway, downregulation of transforming growth factor beta 1 expression and alleviation of perifollicular inflammation. These effects collectively regulated the hair follicle cycle and promoted hair regeneration. Overall, our results highlighted a promising therapeutic approach for AGA with potential translational possibilities.

## Graphical abstract

**Figure:**
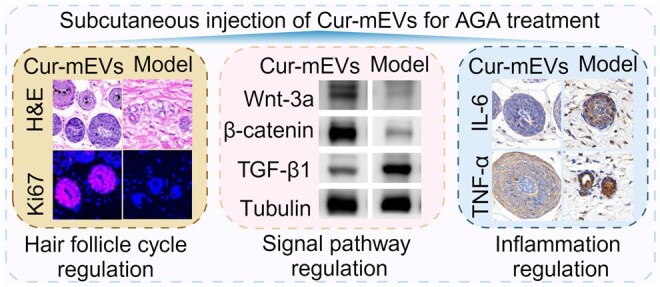

## Introduction

Androgenetic alopecia (AGA) is a global health concern, affecting approximately 50% of middle-aged white men [1]. This condition significantly affects both the patients’ appearance and their social interactions and psychological well-being [2, 3]. To date, only two therapeutics—minoxidil and finasteride—have been approved by the US Food and Drug Administration (FDA) for the treatment of AGA [4]. However, both of them exhibit limited efficacy and are associated with significant side effects [5, 6]. Therefore, the development of novel therapies that effectively promote hair regrowth while ensuring high biosafety are in urgent need. Achieving this goal requires a comprehensive understanding of the regulating mechanism of AGA.

AGA is characterized by upregulated androgen level and inflammation in the hair follicle (HF) microenvironment [7, 8]. Androgens are believed to be the key mediators in the pathogenesis of AGA [9]. Upon binding to their receptors, androgens trigger the ubiquitination and degradation of β-catenin in targeted cells, particularly dermal papilla cells (DPCs). This degradation impairs the association of β-catenin with T-cell factor (TCF) and lymphoid enhancer-binding factor (LEF), preventing the formation of the β-catenin/TCF/LEF complex. As a result, the translocation of this complex into the nucleus is blocked, leading to the suppression of downstream target genes involved in cell proliferation and differentiation, which in turn, inhibits the Wnt/β-catenin signaling pathway [10, 11]. Moreover, androgens upregulate the expression of TGF-β1 by DPCs, and this cytokine is a well-established negative regulator of hair regeneration [12, 13]. Ultimately, these alterations lead to the inhibition of hair follicle stem cells (HFSCs) activation, disruption of the HF cycle and the onset of hair loss [14, 15]. Current clinical treatments for AGA primarily rely on oral finasteride, a 5α-reductase inhibitor that significantly alters systemic androgen levels and exerts anti-androgenic effects [16]. Although long-term use of finasteride can partially alleviate symptoms of AGA, its systemic side effects, including sexual dysfunction, gynecomastia, and psychological effects (e.g. depression and suicidal tendencies) remain unresolved [17]. While some studies have explored alternative formulations of finasteride, such as nanoparticle-based topical delivery, these approaches are still in the experimental or preclinical stages [18, 19]. Inflammation in the HF microenvironment represents another key mechanism in the development of AGA [8]. Numerous studies have shown that chronic perifollicular infiltration by inflammatory cells, such as macrophages and lymphocytes, is a hallmark histological feature of AGA [20–22]. These inflammatory cells secrete pro-inflammatory cytokines, which damage DPCs and HFSCs, disrupting the normal HF cycle [23, 24]. Unfortunately, there are currently no clinically approved drugs that specifically target the inflammation in the HF microenvironment. Only a limited number of laboratory studies have investigated the use of anti-inflammatory agents, delivered topically in nanoparticle formulations, as a potential treatment for AGA [25].

Curcumin, a natural polyphenolic compound derived from turmeric root [26], is well-known for its potent anti-inflammatory properties and has demonstrated therapeutic effects in various inflammation-related conditions [27, 28]. However, its poor solubility and stability in physiological conditions result in low bioavailability, which limits its clinical application [29]. One promising strategy to overcome these limitations is the use of nanoparticle carriers to encapsulate curcumin, thereby enhancing its bioavailability. Among the various nanoparticle carriers, extracellular vesicles (EVs) have emerged as a particularly promising option [30]. EVs are particles secreted by various cell types into the extracellular environment [31]. These vesicles not only carry bioactive drugs but also regulate diverse physiological activities in the body due to their cargo of biologically active substances derived from parent cells [32]. Due to these unique properties, EVs are widely regarded as ideal nanoparticle carriers for therapeutic applications [33–35]. Within this category, milk-derived extracellular vesicles (mEVs) have gained considerable attention in recent years due to their easy accessibility, cost-effectiveness and scalability [36]. Notably, mEVs have demonstrated their strong potential in tissue regeneration, including wound healing, bone regeneration and intestinal regeneration, etc. [37–40]. Moreover, mEVs have been reported to exhibit potent anti-inflammatory effects in various inflammatory diseases, including arthritis, inflammatory bowel disease, and gingivitis [41–43].

**Figure 1.**
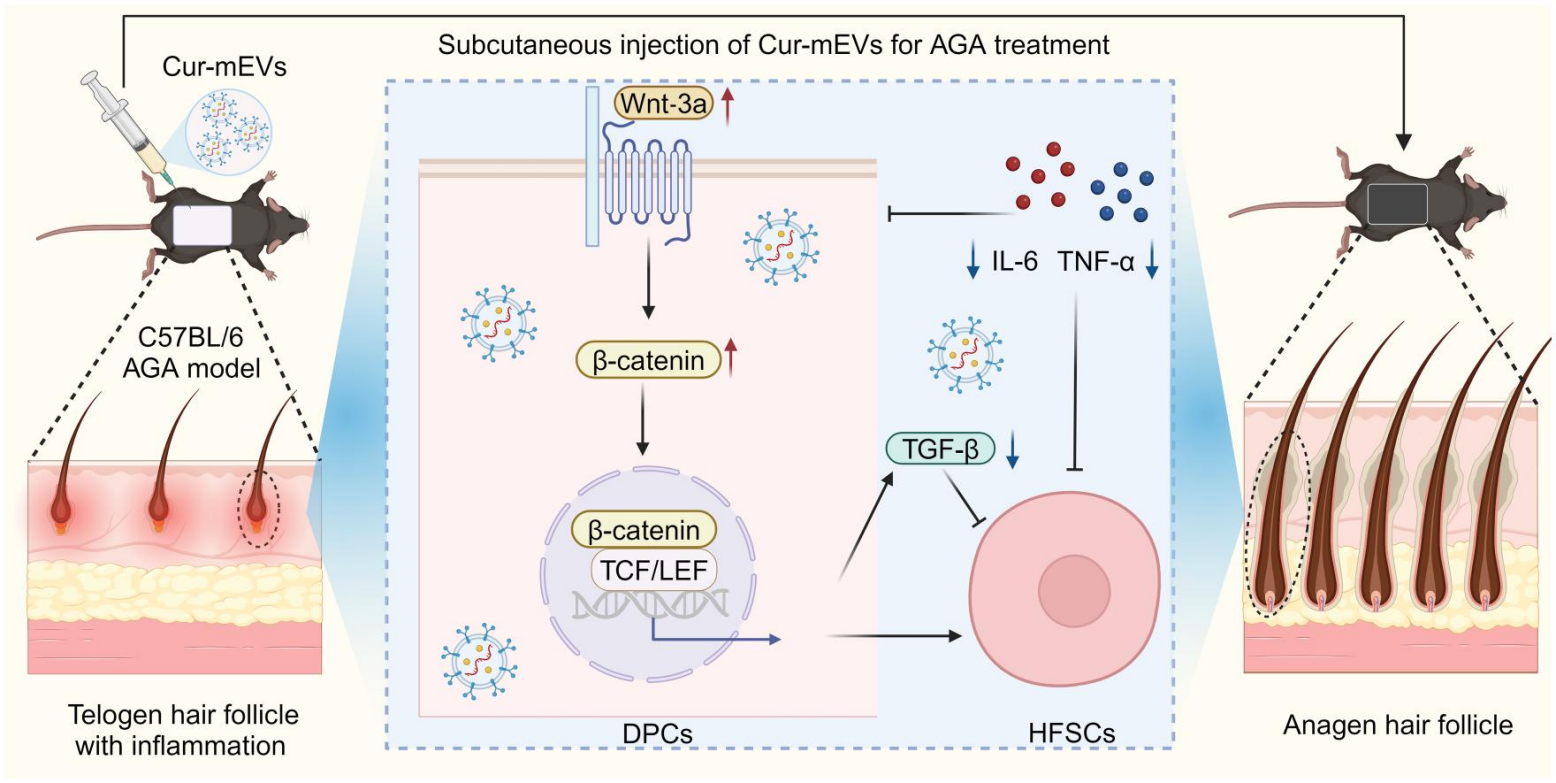
Diagrammatic representation of Cur-mEVs for the treatment of AGA by remodeling HF microenvironment.

Herein, we proposed curcumin-primed mEVs (Cur-mEVs) for the treatment of AGA with remodeling the microenvironment of HF. High-purity mEVs using acetic acid (AA)-assisted ultracentrifugation were initially isolated and loaded with curcumin *via* electroporation to formulate Cur-mEVs. Our findings demonstrated that Cur-mEVs could significantly activate HFSCs in the AGA mouse model by upregulating the Wnt/β-catenin signaling pathway, downregulating TGF-β1 expression, and reducing inflammation in the HF microenvironment, thereby modulating the HF cycle and promoting hair regeneration (as illustrated in Figure 1). We believe that our study offers new perspectives for the treatment of AGA with Cur-mEVs.

## Materials and methods

### Reagents

Defatted bovine milk was sourced from Inner Mongolia Mengniu Dairy (Group) Limited by Share Ltd. Curcumin (HY-N0005) was acquired from MedChemExpress LLC, and testosterone (T8600) from Solarbio Science & Technology Co., Ltd. The study utilized rabbit polyclonal antibodies, including TSG101 (28283), CD9 (20597), CD63 (25682), CD81 (27855), Alix (12422), Calnexin (10427), β-catenin (51067), TGF-β1 (21898) and Tubulin (11224), all obtained from Proteintech Group, Inc.; Ki67 (GB121141), SOX9 (GB14171), IL-6 (GB11117), and TNF-α (GB11188), which were obtained from Servicebio Technology Co., Ltd; Wnt3a (DF6113) was purchased from Affinity Biosciences Group, Ltd.

### Isolation of mEVs

AA was added to defatted bovine milk at a volume ratio of 1:100 (AA:milk). The mixture was stirred for 5 min and then centrifuged at 13 000 g for 1 h at 4°C [44]. The supernatant was filtered using a 0.22-µm membrane to separate the whey. The whey was ultracentrifuged at 130 000 g for 90 min at 4°C with an SW32Ti rotor (Beckman Coulter, USA) to obtain the mEVs pellet. PBS was used to resuspend the pellet, which then underwent a second ultracentrifugation under identical conditions to obtain mEVs.

### Preparation of Cur-mEVs

A 500-µL mixture was prepared by mixing 7.5 µg of curcumin and 160 µg of mEVs in PBS containing 5% trehalose. The mixture was subjected to electroporation using an electroporator (Bio-Rad, USA) in a 4-mm cuvette at 400 V and 150 μF, followed by incubation at 37°C for 30 min. It was then centrifuged at 13 000 rpm for 30 min at 4°C to remove free curcumin precipitates. Finally, the suspension was ultracentrifuged at 130 000 g for 90 min at 4°C to isolate the Cur-mEVs pellet, which was resuspended in PBS to yield Cur-mEVs.

### Characterizations of mEVs and Cur-mEVs

Transmission electron microscopy (TEM, JEM-1200EX, Japan) was used to observe the morphological features of mEVs and Cur-mEVs. The Zetaview PMX120-Z system (Particle Metrix, Germany) was employed for nanoparticle tracking analysis (NTA) to measure their particle size distribution and concentration. Western blotting (WB) was employed to verify the presence of characteristic EV protein markers, including TSG101, CD9, Alix, CD63, CD81, and Calnexin, in the purified vesicles. The protein concentration was determined using the Bicinchoninic Acid （BCA) assay. The drug loading efficiency and recovery rate of mEVs were calculated using the following formula: 


$$\begin{array}{l} \text{Encapsulation}\;\text{efficiency}\;\left(\text{EE},\%\right)=M_{\text{loading}}\;/\;M_{\text{Cur}}\times 100\%\\ \text{Drug}\;\text{loading}\;（\%）=M_{\text{loading}}\;/\;\left(M_{\text{loading}}+M_{\text{mEVs}}\right)\times 100\%\\ \text{Recovery}\;\text{rate}\;\text{of}\;\text{mEVs}\;(\%)=M_{\text{mEVs},\text{after}}\;/\;M_{\text{mEVs}}\times 100\% \end{array}$$

where M_loading_ represents the amount of curcumin loaded into mEVs; M_Cur_ is the initial amount of curcumin added to the reaction system; M_mEVs_ is the initial amount of mEVs; M_mEVs,after_ refers to the amount of mEVs after drug loading.

### Real-time quantitative polymerase chain reaction

Skin tissue total RNA was extracted, and its concentration and purity were evaluated using a NanoDrop-2000 spectrophotometer (Thermo Fisher Scientific, USA). RNA was reverse-transcribed into cDNA using HiScript II Q RT SuperMix (R223, Vazyme), and subsequently, Real-Time Quantitative Polymerase Chain Reaction (RT-qPCR) was conducted with the SYBR Prime Script kit (Q711, Vazyme). Results were normalized using GAPDH as the internal control. The target gene primer sequences were designed as follows:

mGAPDH, 5′-GAAGGTCGGTGTGAACGGATTTG-3′ (sense)
5′-CATGTAGACCATGTAGTTGAGGTCA-3′ (antisense)
mWnt3a, 5′-AACTGCACCACCGTCAGCAACA-3′ (sense)
5′-AGCGTGTCACTGCGAAAGCTA-3′ (antisense)
mβ-catenin, 5′-GCTGGGACCCTTCACAACCTT-3′ (sense)
5′-GCTGGGATGCCACCAGACTTA-3′ (antisense)
mTGF-β1, 5′-CTCCCGTGGCTTCTAGTGCTGG-3′ (sense)
5′-GCTTCGATGCGCTTCCGTTTCA-3′ (antisense)

### Western blot

Proteins from skin tissue were analyzed using SDS-PAGE and transferred to PVDF membranes. Membranes were incubated overnight at 4°C with primary antibodies targeting Wnt3a (1:1000), β-catenin (1:5000), TGF-β1 (1:5000) and Tubulin (1:5000). After washing, HRP-conjugated secondary antibody (SA00001, Proteintech) was applied. Protein signals were detected using a Bio-Rad chemiluminescence imaging system. Tubulin was used as the endogenous control.

### Establishment of AGA mouse model

In this study, topical testosterone of three different doses for AGA mouse model establishment were carefully investigated. Seven-week-old male C57BL/6 mice were obtained from Zhuhai BesTest Bio-Tech Co., Ltd. Animal studies followed protocols approved by the Institutional Animal Care and Use Committee and the Laboratory Animal Welfare and Ethics Committee of Sun Yat-sen University (SYSU-IACUC-2024-000618).

A 6 cm^2^ (2 cm × 3 cm) depilated area was prepared on the dorsal skin of the mice, which were subsequently randomly divided into four groups: Blank, Low-dose, Mid-dose, and High-dose. Three groups, excluding the Blank group, were administered daily topical applications of a 0.5% (w/v) testosterone solution in 50% (v/v) ethanol at doses of 50, 100 and 200 µL/cm^2^, respectively, beginning the first day after depilation and continuing for 28 days. Mice were photographed on days 1, 8, 13, 16, 19, 22, 25 and 28 to observe alterations in skin color and hair regrowth. On days 13 and 28, skin samples were collected for further analysis. On day 28, the FotoFinder Digital Trichoscope (FotoFinder Systems GmbH, Germany) was used to measure the diameter and density of regenerated hair. And the regenerated hair’s surface structure was examined and imaged with a Thermo Fisher Apreo 2C scanning electron microscope (SEM) featuring an Oxford ULTIM Max65 energy-dispersive X-ray spectroscopy system. For the animal experiment involving Cur-mEVs treatment for AGA, the high-dose testosterone protocol was selected for model establishment. The procedure involved creating an 8 cm^2^ (2 cm × 4 cm) depilated area on the dorsal skin of the mice, after which the mice were randomly divided into five groups: Model, Curcumin, mEVs, Cur-mEVs, and Blank. Apart from Blank group, the remaining four groups received the high-dose protocol, starting on the first day post-depilation and continuing for 27 consecutive days. Photographs of mice were taken on days 1, 9, 12, 15, 18, 21, 24 and 27 to record changes in skin color and hair growth in the depilated region. Additionally, skin samples were collected on days 15 and 27 for further experimental analysis.

### Therapeutic evaluation of Cur-mEVs in the established AGA mouse model

To evaluate the therapeutic efficacy of Cur-mEVs, AGA mice received subcutaneous injections of curcumin (250 µg/mL, 200 µL), mEVs (2000 µg/mL, 200 µL) and Cur-mEVs (with dosages of each component matching those in the Curcumin and mEVs groups) at six designated points on the corresponding dorsal skin. Treatments were given on alternate days from the day after depilation until day 13. The sample collection and analysis procedures were the same as the description above.

### Biodistribution and biocompatibility of Cur-mEVs

The *in vivo* distribution of Cur-mEVs was evaluated in the Free-DiR and DiR-Cur-mEVs groups. The free-DiR group received subcutaneous multi-point injections of free-DiR (5 µg) into the shaved dorsal skin, while the DiR-Cur-mEVs group received DiR-Cur-mEVs (Cur-mEVs protein concentration: 2000 µg/mL; DiR: 5 µg) under identical conditions. The injection volume for both groups was 200 μl. Skin samples from the shaved area, along with major organs, were collected at 4, 24 and 48 h post-injection following the sacrifice of the mice. Fluorescence intensity in the skin and organs was measured using In Vivo Imaging System (IVIS, PerkinElmer, USA) to assess the distribution and retention of the injected compounds. For the evaluation of *in vivo* safety, the grouping and treatment of mice are described in the above sections, which detail the therapeutic experiments. Body weight was monitored throughout the treatment period. Serum was collected on day 27 to measure levels of ALT, AST, CREA and BUN. H&E staining was performed on the collected organs to assess the biosafety and biocompatibility of Cur-mEVs.

### Histological staining of skin samples

In model establishment experiment, skin samples were collected from mice on day 13 for H&E staining and immunofluorescence (IF) analysis, with the latter employed to assess Ki67 expression. On day 28, the remaining mice were sacrificed for subsequent H&E staining. In the therapy study, mice were euthanized on day 15 for H&E staining and evaluation of Ki67, SOX9, IL-6 and TNF-α levels in the depilated area using IF or immunohistochemistry (IHC). On day 27, the remaining mice were sacrificed for H&E staining. HF cycle phases were identified based on the histological features of HFs observed in H&E-stained sections [45]. Scores were assigned to each phase, and the number of follicles in each phase was multiplied by the corresponding score. The products were then summed and divided by the total number of follicles to calculate the HF cycle score [46, 47]. The detailed scoring tables are provided in Supplementary Table S1. For IF and IHC staining, the primary antibodies used included those targeting Ki67 (1:300), SOX9 (1:200), IL-6 (1:1000) and TNF-α (1:600).

### Statistical analysis

Data are presented as mean ± standard deviation. Group differences were evaluated using one-way analysis of variance. Statistical significance was determined using the following thresholds: *P < 0.05, **P < 0.01, ***P < 0.001.

## Results

### Preparation and characterizations of Cur-mEVs

**Figure 2.**
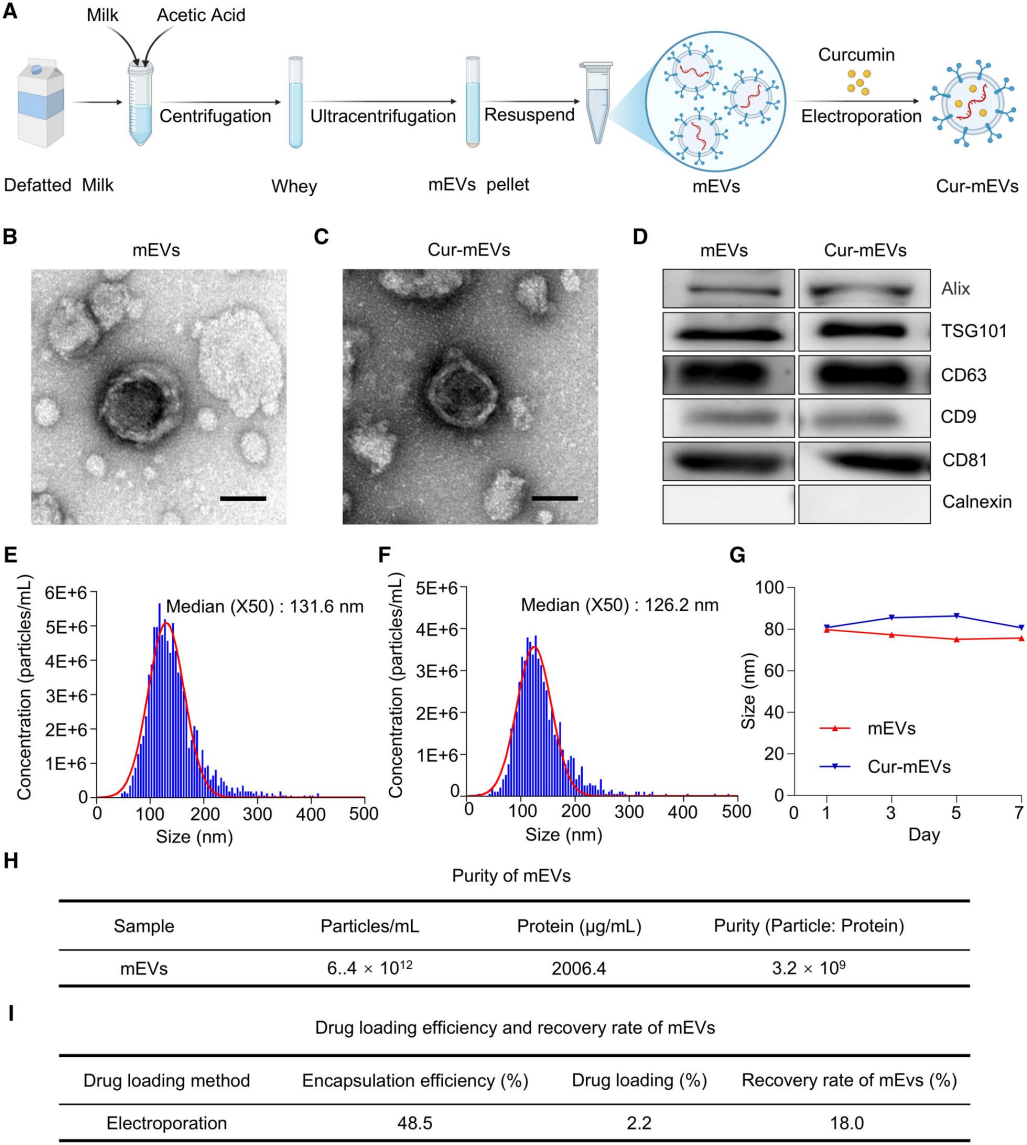
Preparation and characterizations of Cur-mEVs. (**A**) Diagrammatic representation of the process for preparing mEVs and Cur-mEVs. (**B** and **C**) TEM of mEVs and Cur-mEVs, scale bar = 50 nm. (**D**) Protein markers of mEVs and Cur-mEVs were characterized using western blot analysis. (**E**) Size distribution of mEVs *via* NTA. (**F**) Size distribution of Cur-mEVs *via* NTA. (**G**) Size of mEVs and Cur-mEVs *via* Malvern Particle Size Analyzer for 7 days. (**H**) Purity of mEVs. (**I**) Drug loading efficiency and recovery rate of mEVs.

mEVs were purified from defatted bovine milk through acid precipitation and subsequent ultracentrifugation. Curcumin was subsequently incorporated into the mEVs *via* electroporation to obtain Cur-mEVs (Figure 2A). The morphologies of both mEVs (Figure 2B) and Cur-mEVs (Figure 2C) were characterized by TEM, revealing that both samples displayed the typical vesicular structures. WB analysis showed positive markers (Alix, TSG101, CD63, CD9, CD81) and absence of calnexin, validating mEV and Cur-mEV identity (Figure 2D). NTA indicated that the median diameters (X50) of mEVs and Cur-mEVs were 131.6 nm (Figure 2E) and 126.2 nm (Figure 2F), respectively. The sizes of mEVs and Cur-mEVs were monitored at 4°C for 7 days using a Malvern Particle Size Analyzer, demonstrating that both EVs exhibited high stability (Figure 2G). The purity of mEVs was determined to be 3.2 × 10^9^ particles/µg protein (Figure 2H), indicating their suitability for subsequent applications. Regarding curcumin loading efficiency, the encapsulation efficiency was calculated as 48.5%, with a drug loading capacity of 2.2%. Additionally, the recovery rate of mEVs, assessed *via* BCA protein assay, was 18.0% (Figure 2I). Collectively, the above results confirm the successful preparation of Cur-mEVs.

### Establishment of AGA mouse model

**Figure 3.**
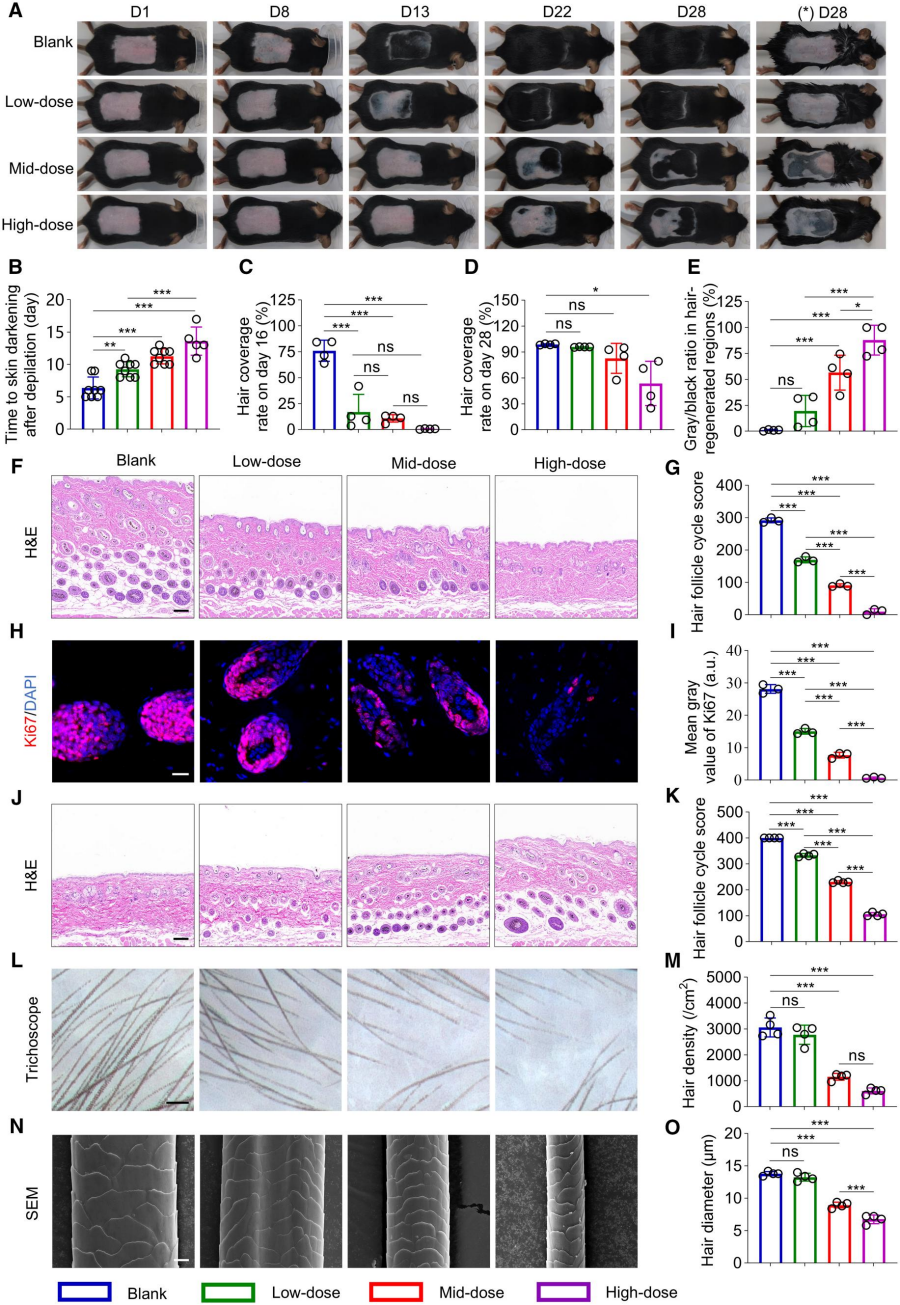
Development of AGA mouse model. (**A**) Representative photographs on day 1, 8, 13, 22 and 28. (*) D28 represents depilation again on day 28. (**B**) Time to skin darkening after depilation. *n* = 5–8. (**C**) Hair coverage rate on day 16. *n* = 4. (**D**) Hair coverage rate on day 28. *n* = 4. (**E**) The proportion of gray/black areas in hair-regenerated regions on day 28. *n* = 4. (**F**) The transition rate of hair follicle on day 13, scale bar = 100 µm. (**G**) Scoring of hair follicle cycle on day 13. *n* = 3. (**H**) IF staining of Ki67 on day 13, scale bar = 20 µm. (**I**) Ki67 fluorescence intensity in each group on day 13. *n* = 3. (**J**) The transition rate of hair follicles on day 28, scale bar = 100 µm. (**K**) Scoring of hair follicle cycle for each group on day 28. *n* = 4. (**L**) Trichoscopic imaging on day 28, scale bar = 100 µm. (**M**) Density of newly grown hair in each group on day 28. *n* = 4. (**N**) SEM imaging of newly grown hair on day 28, scale bar = 2 µm. (**O**) Diameter of newly grown hair in each group on day 28. *n* = 20. ns, nonsignificant (P > 0.05), *P < 0.05, **P < 0.01, ***P < 0.001.

The HF cycle primarily includes three phases: anagen, catagen and telogen [48]. During a complete HF cycle, three dynamic changes associated with regenerated hair can be observed in the depilated skin area [45]: (i) skin color shifts from pink to black, marking the transition from telogen to anagen phase; (ii) the emergence of new hair shafts above the skin surface; and (iii) skin color reverts from black/gray to pink, indicating the transition from anagen/catagen back to telogen phase. Currently, there is no universally accepted method or standardized criteria for the successful development of the AGA mouse model [47]. In most existing studies, the success of AGA model is typically evaluated based solely on delayed skin darkening [49]. However, other critical aspects of the complete HF cycle as described above are often underestimated. Therefore, we believe that a successful AGA mouse model should be evaluated using a more comprehensive and multidimensional set of criteria. Thus, in this study, the impact of different doses (low, mid and high) of topical testosterone on the development of the AGA mouse model were investigated with the above processes monitored by direct images (Figure 3A), measuring the time to skin darkening, hair coverage rate, and the proportion of gray/black areas in hair-regenerated regions.

Regarding the time to skin darkening, the three treatment groups showed significantly longer darkening time compared to the Blank group, indicating that all three doses of topical testosterone effectively delayed the initiation of hair regeneration. Moreover, the High-dose group exhibited a significantly longer darkening time than the other groups (Figure 3B), suggesting a dose-dependent inhibitory effect of testosterone on hair regeneration. Until day 13 post-depilation, no significant differences in hair coverage rate were observed among the groups (Supplementary Figure S1), with differences observed from day 16 that all three treatment groups showed significantly lower hair coverage rate compared to the Blank group (Figure 3C). In the following experimental periods, all groups showed a gradual increase in hair coverage rate, with Low-dose and Mid-dose groups approaching a similar status to the Blank group (Supplementary Figure S1). By day 28, only the High-dose group exhibited significantly lower hair coverage rate compared to the Blank group (Figure 3D). Following repeated depilation on day 28 (experimental end point), it was observed that the skin color of the Blank group had largely reverted to pink, with gray/black area proportion being only 1.1 ± 0.7%. In contrast, the Mid-dose and High-dose groups still maintained a significant proportion of gray/black area, with 56.4 ± 16.8% and 87.8 ± 14.4%, respectively, which were significantly higher than that of the Blank group (Figure 3E), suggesting that a substantial proportion of the newly grown hair in these two groups remained in the anagen or catagen phase.

The HFs were histologically examined by H&E staining on day 13 post-depilation, revealing that they had fully entered the anagen phase in the Blank group, while in the High-dose group, almost all HFs remained in the telogen phase (Figure 3F). The transition from telogen to anagen typically involves an increase in the thickness of the skin and subcutaneous layer [50]; therefore, this parameter was also carefully evaluated. The thickness decreased with the increase of testosterone dosage, and the High-dose group displayed significantly lower thickness than the other groups (Figure 3F and Supplementary Figure S2A). To further evaluate the HF cycle distribution, follicular cycle scores were applied, with the High-dose group showing the lowest score (9.7 ± 8.6) (Figure 3G). Similarly, on day 13, the expression of Ki67 (a cell proliferation marker [51]) in the HFs of the High-dose group was the lowest (Figure 3H and I), indicating that this group most significantly inhibited the cell proliferation process associated with the transition of the HF cycle. These results indicated that the High-dose group demonstrated the most effective inhibition of the transition of HFs from the telogen phase to the anagen phase. On day 28, it revealed that all HFs of the skin tissue in the Blank group had returned to the telogen phase, while in the High-dose group, they remained primarily in the anagen phase (Figure 3J). Additionally, the skin and subcutaneous layers were markedly thicker in the High-dose group than in the Blank group (Supplementary Figure S2B). The histological features in the Low-dose and Mid-dose groups were found to in between (Figure 3J and Supplementary Figure S2B). Furthermore, the HF cycle score in the High-dose group was the lowest (105.3 ± 6.8), significantly lower than that of the other three groups (Figure 3K). This suggested that the High-dose group most significantly inhibited the transition of HFs from anagen back to telogen.

The hair density was characterized using a trichoscope as shown in Figure 3L and M with both Mid-dose and High-dose groups exhibited a significant decrease in comparison to the Blank group. Furthermore, the morphologies of the regenerated hair were imaged *via* SEM, which displayed that the cuticle scales of the hair in the Mid-dose and High-dose groups were smaller and damaged compared to those in the Blank and Low-dose groups (Figure 3N). Additionally, the diameter of the regenerated hair was also significantly reduced in the High-dose group (Figure 3O).

Briefly, the above results illustrated that the High-dose group exhibited the longest time to skin darkening, lowest hair coverage rate, highest proportion of gray/black areas and the most significant reduction in hair density and diameter, indicating the High-dose group most significantly delayed the transition of HFs from telogen to anagen, inhibited the speed of new hair emergence from the skin surface with persistent and disrupted the morphological characteristics of new hair. Taken together, these results suggested that the High-dose group provided multidimensional criteria for the successful development of the AGA mouse model, while demonstrating the most robust model development effect.

### Cur-mEVs promote hair regeneration in the AGA mouse model

**Figure 4.**
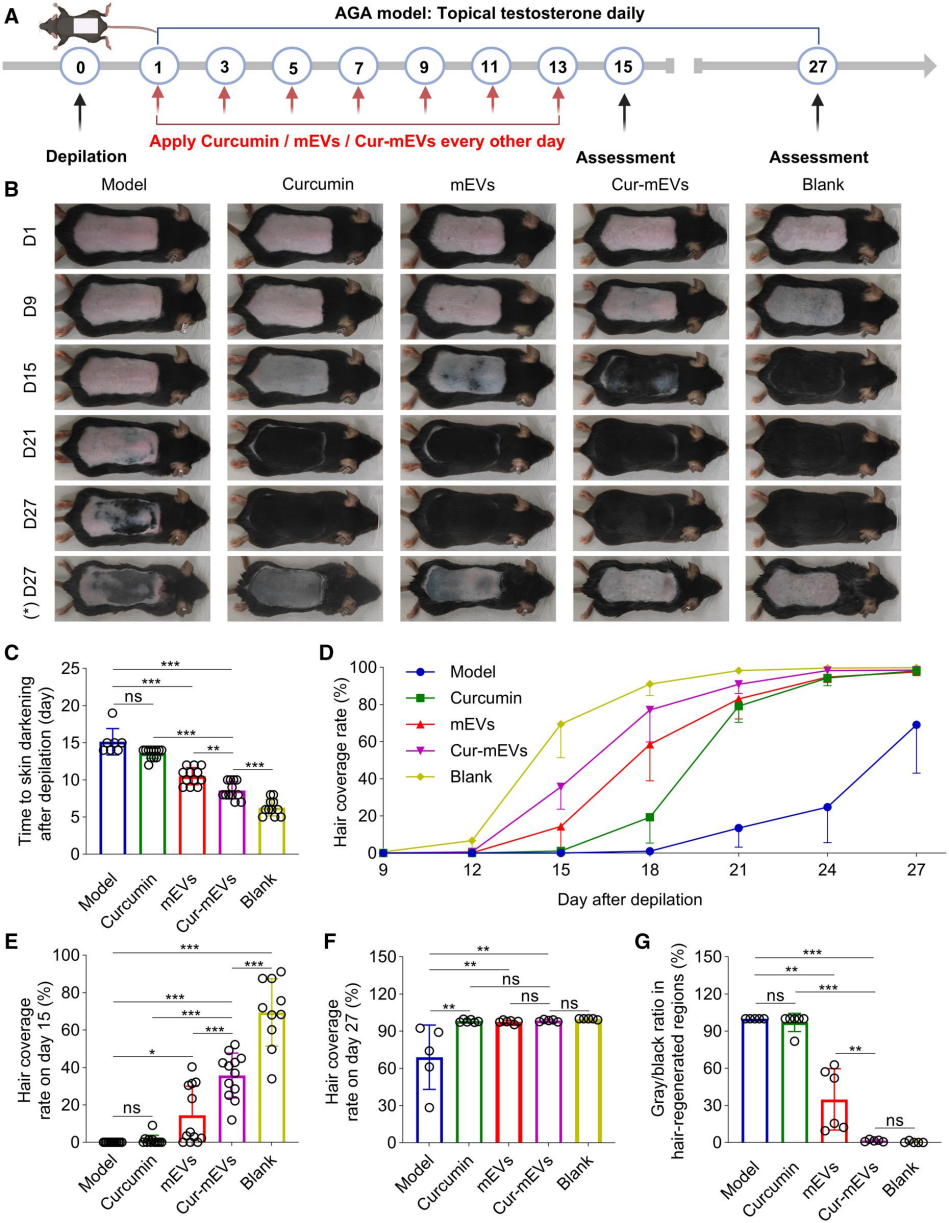
Cur-mEVs promote hair regeneration in the AGA mouse model. (**A**) Diagrammatic representation of AGA mouse model and group-specific treatments. (**B**) Representative photographs on day 1, 9, 15, 21 and 27. (*) D27 represents depilation again on day 27. (**C**) Time to skin darkening after depilation. *n* = 7-12. (**D**) Hair coverage rate at different time points after depilation. (**E**) Hair coverage rate on day 15. *n* = 11-12. (**F**) Hair coverage rate on day 27. *n* = 5-6. (**G**) The proportion of gray/black areas in hair-regenerated regions on day 27. *n* = 5-6. ns, nonsignificant (P > 0.05), *P < 0.05, **P < 0.01, ***P < 0.001.

Hair regeneration efficiency was evaluated through direct images and several quantitative indicators, including the time to skin darkening, hair coverage rate, and the proportion of gray/black areas, to assess the therapeutic efficiency of Cur-mEVs in the AGA mouse model (Figure 4A and B).

In the Model group, the time to skin darkening (15.1 ± 1.7 days) was significantly prolonged compared to the Blank group (6.3 ± 1.1 days), confirming that testosterone application effectively delayed hair regeneration, thus validating successful modeling. Among the experimental groups, the Cur-mEVs group exhibited the shortest time to skin darkening (8.6 ± 1.1 days), which was significantly shorter than that of the Model group, indicating a stronger reversal of testosterone’s inhibitory effects on hair regeneration compared to the Curcumin and mEVs groups (Figure 4C).

Regarding hair coverage, no significant difference was observed among the groups until day 12 post-depilation (Figure 4D). However, from day 15, the Model and Curcumin groups exhibited minimal hair growth, with coverage rates of 0% and 1.1 ± 2.6%, respectively. In contrast, the Cur-mEVs group achieved a significantly higher coverage rate of 35.7 ± 12.0%, which was closest to that of the Blank group (69.4 ± 18.0%), while the mEVs group demonstrated moderate improvement (14.3 ± 15.7%) (Figure 4E), highlighting the superior hair regeneration of the Cur-mEVs group compared to other groups. Throughout the study, the Model group consistently exhibited the lowest hair coverage rate, reaching only 69.0 ± 26.0% by day 27. From day 21 onward, the other groups showed convergence in hair coverage rate, approaching nearly 100% by day 27 (Figure 4F).

Following repeated depilation, the skin color of the Cur-mEVs group returned to pink, which was similar to the Blank group, with gray/black area proportions of 1.5 ± 0.8% and 0.4 ± 0.8%, respectively, indicating that most hair had reverted to the telogen phase. In contrast, the Curcumin and Model groups retained predominantly gray/black skin, with gray/black area proportions of 97.0 ± 7.4% and 100%, respectively, suggesting that hair remained primarily in the anagen or catagen phase. The mEVs group exhibited partial reversion to pink, with a gray/black proportion of 34.9 ± 24.9%, reflecting a partial return to the telogen phase (Figure 4G).

Collectively, Cur-mEVs induced the fastest skin darkening, achieved the highest hair coverage rate in the shortest time, and showed the lowest proportion of gray/black areas in newly regenerated hair compared to other treatment groups. These results suggested that Cur-mEVs most efficiently counteracted the suppression of hair regeneration induced by model establishment, demonstrating the strongest therapeutic efficacy.

### Cur-mEVs accelerates the transition of the HF cycle

HFs exhibit distinct histological features at different stages of the HF cycle under H&E staining. According to the morphology of the hair bulb, its layer position, and the location of the distal end of the newly regenerated hair shaft, the anagen phase can be divided into early (I–IIIa), mid (IIIb–IIIc) and late (IV–VI) sub-phases. Similarly, the catagen phase is classified into early catagen (I–IV), mid-catagen (V–VI) and late catagen (VII–VIII) [45].

**Figure 5.**
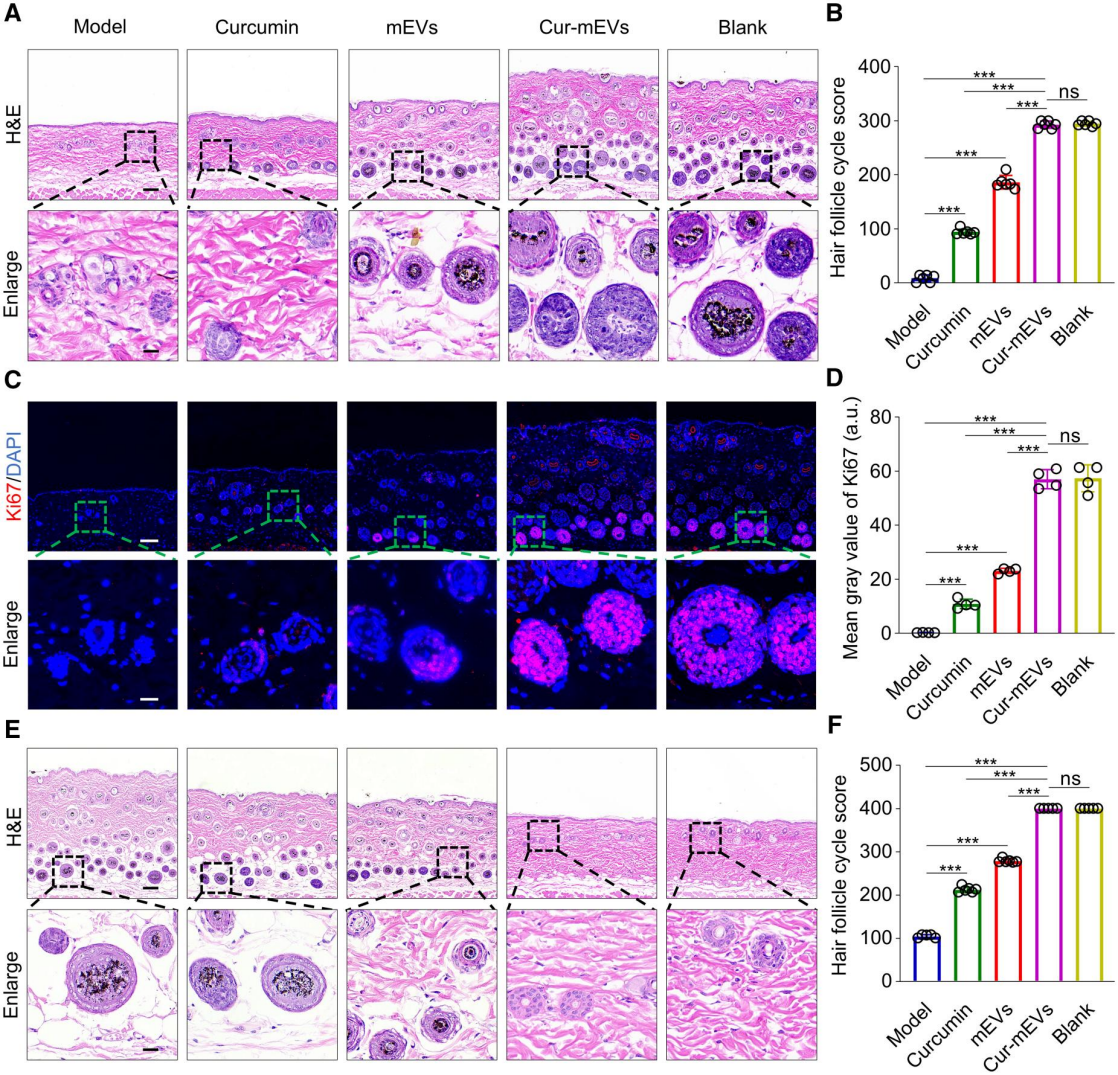
Cur-mEVs accelerate the transition of the hair follicle cycle. (**A**) The transition rate of hair follicles on day 15, scale bar = 100 µm (enlarged view: 20 µm). (**B**) Hair follicle cycle score of the skin on day 15. *n* = 6. (**C**) IF staining of Ki67 on day 15, scale bar = 100 µm (enlarged view: 20 µm). (**D**) Ki67 fluorescence intensity on day 13. *n* = 4. (**E**) The transition rate of hair follicle on day 27, scale bar = 100 µm (enlarged view: 20 µm). (**F**) Hair follicle cycle score on day 27. *n* = 5–6. ns, nonsignificant (P > 0.05), *P < 0.05, **P < 0.01, ***P < 0.001.

The role of Cur-mEVs in HF cycle regulation was further investigated by H&E staining of skin samples on day 15. In the Model group, most HFs were arrested in the telogen phase, characterized by miniaturized follicles located at the level of the dermal sebaceous glands. In contrast, the Cur-mEVs group, similar to the Blank group, predominantly displayed HFs in the late anagen phase, distinguished by enlarged, densely packed hair bulbs situated deep in the subcutaneous tissue and substantial new hair shafts emerging from the skin surface. The Curcumin group showed that follicles had entered the early anagen, with moderately dense, enlarged hair bulbs located in the dermal–subcutaneous junction. The mEVs group exhibited follicles in the mid-anagen, with histological features intermediate between those of the Curcumin and Cur-mEVs groups (Figure 5A). The corresponding follicle cycle scoring revealed that the Cur-mEVs group exhibited the highest score, comparable to the Blank group (292.8 ± 6.7 vs. 294.8 ± 4.6). The Curcumin group displayed a higher score than the Model group (94.3 ± 5.4 vs. 8.2 ± 6.7), indicating a moderate regulatory effect, while the mEVs group scored an intermediate 186.3 ± 12.5 (Figure 5B). Moreover, the Cur-mEVs group demonstrated significantly greater skin and subcutaneous thickness compared to the Model group (826.5 ± 35.8 μm vs. 405.7 ± 12.6 μm) (Supplementary Figure S3A). The Cur-mEVs group also exhibited the highest Ki67 expression (Figure 5C), which was comparable to the Blank group (57.1 ± 3.5 vs. 57.4 ± 5.0), indicating active cell proliferation associated with follicle cycle transition (Figure 5D).

On day 27, it showed that in the Model group, most HFs remained in the anagen or early catagen phases, characterized by enlarged, dense hair bulbs located in the deep subcutaneous tissue in H&E staining. In contrast, the Cur-mEVs group, similar to the Blank group, had most follicles in the telogen phase, marked by low-density, shrunken follicles at the level of the dermal sebaceous glands. In the mEVs group, follicles were primarily in the late catagen phase, exhibiting shrinking bulbs at the dermal–subcutaneous junction, whereas follicles in the Curcumin group were in the mid-catagen phase, showing characteristics between those of the mEVs and Model groups (Figure 5E). During the anagen/catagen-to-telogen transition, a significant reduction in skin and subcutaneous thickness was observed, with the Cur-mEVs group displaying a notable decrease compared to the Model group (430.9 ± 34.0 μm vs. 808.1 ± 54.9 μm) (Supplementary Figure S3B). Consistent with these findings, the Cur-mEVs group achieved the highest follicle cycle score, comparable to the Blank group (Figure 5F), confirming its pivotal role in facilitating the anagen-to-telogen transition.

### Cur-mEVs modulate the Wnt/β-catenin signaling pathway and TGF-β1 expression

**Figure 6.**
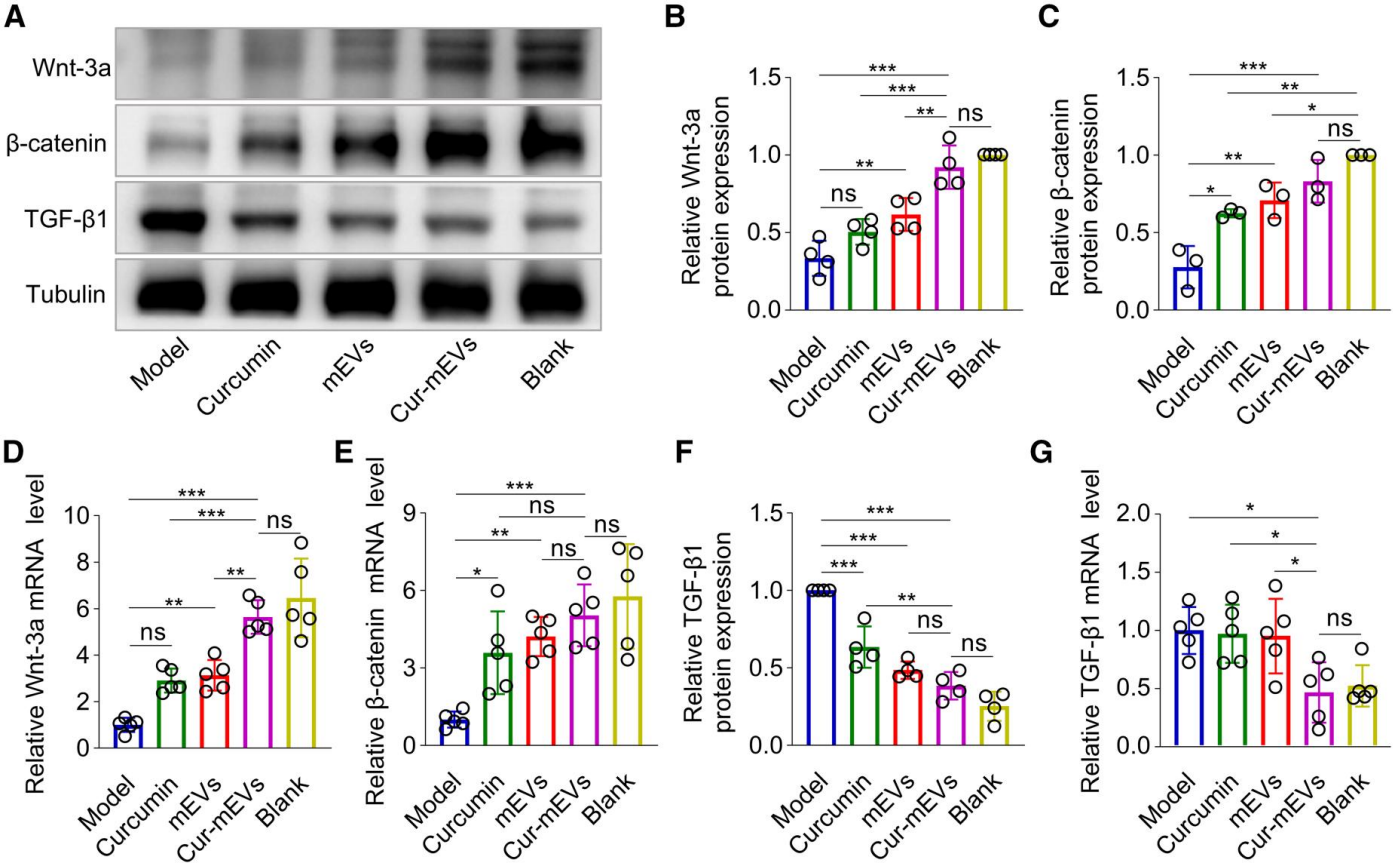
Cur-mEVs modulate the Wnt/β-catenin signaling pathway and TGF-β1 expression. (**A**) Protein levels of Wnt-3a, β-catenin and TGF-β1 in each group on day 15. (**B**) Relative protein expression of Wnt-3a on day 15. n = 4. (**C**) Relative protein expression of β-catenin on day 15. *n* = 3. (**D** and **E**) Relative mRNA levels of the Wnt-3a, β-catenin on day 15. *n* = 5. (**F**) Relative protein expression of TGF-β1 on day 15. *n* = 4. (**G**) Relative mRNA levels of TGF-β1 on day 15. *n* = 5. ns, nonsignificant (P > 0.05), *P < 0.05, **P < 0.01, ***P < 0.001.

The Wnt/β-catenin signaling pathway plays a key role in regulating the HF cycle, particularly in the transition from telogen to anagen [52, 53]. TGF-β1, in contrast, has been identified as a prominent suppressor of hair regeneration [13]. To assess the effect of Cur-mEVs on these signaling pathway and molecule, skin samples from depilated areas were collected on day 15 for WB and qPCR analysis. As shown in Figure 6A, the mEVs and Cur-mEVs significantly upregulated Wnt-3a protein levels by 1.7- and 2.6-fold, and β-catenin levels by 2.5- and 3.0-fold, respectively (Figure 6B and C) as compared to the Model group. To further assess the expression of Wnt-3a and β-catenin at the mRNA level, qPCR analysis was performed. The Curcumin, mEVs, and Cur-mEVs groups elevated Wnt-3a mRNA levels by 2.9-, 3.1-, and 5.6-fold, and β-catenin mRNA levels by 3.6-, 4.2-, and 5.0-fold, respectively, compared to the Model group (Figure 6D and E). These findings indicated that Cur-mEVs treatment exerted a more pronounced effect on both protein and mRNA expression levels of Wnt-3a and β-catenin, closely resembling the levels observed in the Blank group. Additionally, the relative protein levels of TGF-β1 as characterized by WB (Figure 6F) were reduced by 1.6-, 2.1-, and 2.6-fold following treatment with curcumin, mEVs, and Cur-mEVs, respectively, compared to the Model group. Notably, the Cur-mEVs group significantly downregulated TGF-β1 mRNA levels by 2.1-fold compared to the Model group, while no significant differences in TGF-β1 mRNA expression were observed among the Curcumin, mEVs, and Model groups (Figure 6G).

### Cur-mEVs reduce inflammation in the perifollicular microenvironment and activate HFSCs

To evaluate the impact of the Cur-mEVs on regulating the inflammation in perifollicular microenvironment and activating HFSCs, skin tissues from the depilated regions were harvested on day 15 for IHC and IF analysis.

**Figure 7.**
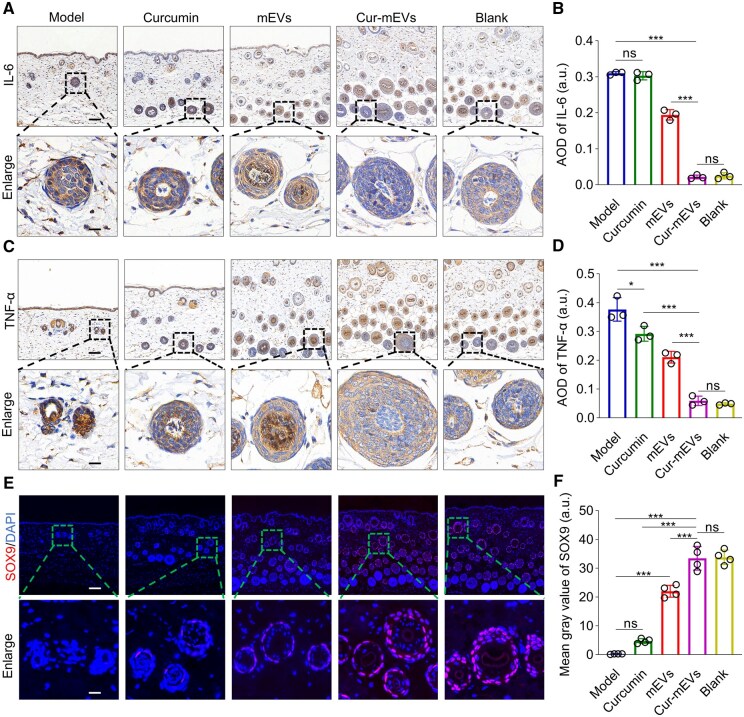
Cur-mEVs reduce inflammation in the perifollicular microenvironment and activate HFSCs. (**A**) IHC staining of IL-6 on day 15, scale bar = 100 µm (enlarged view: 20 µm). (**B**) Relative expression of IL-6 in each group on day 15. *n* = 3. (**C**) IHC staining of TNF-α on day 15, scale bar = 100 µm (enlarged view: 20 µm). (**D**) Relative expression of TNF-α in each group on day 15. *n* = 3. (**E**) IF staining of SOX9 on day 15, scale bar = 100 µm (enlarged view: 20 µm). (**F**) SOX9 fluorescence intensity on day 15. *n* = 4. ns, nonsignificant (P > 0.05), *P < 0.05, **P < 0.01, ***P < 0.001.

Compared to the Model group, the Cur-mEVs group significantly reduced IL-6 and TNF-α levels, which were similar to those observed in the Blank group. The mEVs group demonstrated a notable decrease in these inflammatory cytokines, while the Curcumin group showed very limited effect (Figure 7A–D). These findings suggested that the Cur-mEVs group exerted the most effective reduction on perifollicular inflammation. Consistent result was found with IF analysis, which revealed that the Cur-mEVs group exhibited the highest expression of SOX9, a key marker of HFSCs [54], compared to the Model group. Although the Curcumin and mEVs groups showed moderate increases in SOX9 expression, their signals were significantly weaker than those observed in the Cur-mEVs group (Figure 7E and F). These results indicated that the Cur-mEVs group was substantially more effective in activating HFSCs compared to either curcumin or mEVs alone.

### *In vivo* biodistribution and biocompatibility evaluation of Cur-mEVs

To study the *in vivo* distribution of Cur-mEVs, mice were subcutaneously injected with DiR-labeled Cur-mEVs, using an equivalent dose of free-DiR as a control. In Supplementary Figures S4 and S5, the DiR-Cur-mEVs group exhibited a fluorescence intensity that was significantly greater than the control at 4, 24, and 48 h after injection, showing increases of 2.4-, 2.6- and 2.2-fold, respectively. These results suggested that DiR-labeled Cur-mEVs achieved superior drug accumulation in the shaved skin area and exhibited prolonged local drug retention compared to free-DiR. We further comprehensively evaluated the biosafety and biocompatibility of Cur-mEVs using multiple approaches. Throughout the treatment period, body weight in all groups showed a gradual and steady increase, with no statistically significant differences observed (Supplementary Figure S6). Furthermore, as shown in Supplementary Figure S7, serum markers of liver and kidney function were comparable across groups, and no significant histopathological changes were detected in organ samples (Supplementary Figure S8). These findings collectively demonstrated the excellent biosafety and biocompatibility of Cur-mEVs in AGA mice.

## Discussion

HFs are miniature organs composed of various cell types, including two stem cell populations with regenerative and differentiation potential: DPCs and HFSCs. As the signaling hub for follicular growth, DPCs play a pivotal role in hair growth and morphogenesis [55]. Additionally, the activation of HFSCs is a critical step in initiating hair regeneration by transitioning HF from the telogen phase to the anagen phase [56, 57]. Through autocrine or paracrine signaling of various molecules, DPCs and the surrounding epithelial matrix cells (primarily HFSCs) interact to form a complex regulatory network [58, 59]. This network, comprising diverse cell types, signaling molecules, extracellular matrix components, and other factors, collectively forms the HF microenvironment, which plays a critical role in regulating the cyclic transitions of the HF and hair regeneration [60].

The therapeutic option for AGA is very limited and the animal model is not well established for research purposes. We selected 7-week-old C57BL/6 mice for modeling because at this age, their HFs are synchronized in the telogen phase. Under stimulating factors such as depilation, their HFs can be induced to transition from telogen to anagen, followed by the spontaneous completion of the entire HF cycle [45, 50]. After exogenous androgen induction, alterations in the natural HF cycle occur, primarily characterized by a suppressed transition rate between cycles and inhibited hair regeneration [61–63]. In this study, we firstly demonstrate the successful development of the AGA mouse model by topical application of high-dose testosterone, which inhibited the transition in the HF cycle, delayed the growth of newly regenerated hair, and altered the morphological characteristics of the new hair. It should be noted that although the high-dose protocol was confirmed effective for modeling through the above multimodal evaluation system, the small sample size does not establish it as a standardized method for AGA mouse modeling. Expanded cohorts and cross-lab validation are needed in the future to establish a more reproducible AGA mouse model. In this study, the high-dose protocol was used in subsequent animal experiments. The results showed that, compared to the Model group, subcutaneous injection of Cur-mEVs exhibited a significantly shorter time to darkening, more effective HF transitions and activation of HFSCs as compared with other groups. We also demonstrate the underlying regulatory mechanisms of Cur-mEVs in the treatment of AGA through upregulating Wnt/β-catenin signaling pathway, downregulating the expression of TGF-β1, as well as significantly reducing the levels of inflammation in the HF microenvironment.

In the field of EV-based treatments for hair loss, there have been a limited number of studies, most of which focus on stem cell-derived EVs [64, 65]. This can be exemplified by a study where EVs derived from MSCs were subcutaneously injected into AGA mouse model daily for 15 consecutive days, resulting in significant hair regeneration [66]. In contrast, our use of mEVs not only achieves effective hair regeneration but also provides significant advantages such as high availability, cost-effectiveness, ease of preparation and scalability for large-scale production [67]. These benefits underscore the promising potential of mEVs for future applications in hair regeneration therapies. Furthermore, to date, only one prior study has reported the use of mEVs in a hair loss mouse model [68]. In this study, the hair loss mouse model was induced by performing depilation on the backs of the mice. They then administered multiple intradermal injections of 200 µg of mEVs every other day for 19 days, inducing accelerated hair regeneration. In comparison, our study is the first to demonstrate their application in the AGA mouse model. Inflammation can directly damage DPCs and HFSCs, disrupting the HF microenvironment, particularly the regulatory network between these two cell types. This disturbance impairs the normal HF cycle, ultimately leading to hair loss [23, 24]. Curcumin, due to its potent anti-inflammatory properties, has demonstrated notable therapeutic effects in various inflammatory-related conditions [69]. Based on this, several studies have explored its potential in treating hair loss [70]. For instance, researchers have demonstrated that curcumin can promote hair regeneration by self-assembling with zinc ions to form curcumin-zinc metal-organic frameworks, which were then incorporated into microneedle patches. When applied to AGA mouse model, these patches exhibited significant hair regeneration effects [63].

The safety of the materials used is also a crucial consideration. Curcumin, derived from turmeric, which has been used as both a spice and a medicinal herb for centuries in various regions around the world [71]. The FDA has recognized curcumin as a generally safe compound and affirmed its non-toxicity [72]. Studies have shown that even daily consumption of up to 12 g of curcumin does not result in harmful effects in humans [73]. And numerous clinical trials and *in vitro/in vivo* laboratory studies have further validated the safety of curcumin [74–76]. Additionally, mEVs, derived from milk, a staple component of the human diet, have consistently demonstrated safety across various studies [77–79]. In our study, no significant differences were observed in body weights or serum biochemical markers of liver and kidney function between the groups. Furthermore, H&E staining of organ sections revealed no apparent morphological changes, further supporting the safety of Cur-mEVs.

In summary, finasteride and minoxidil are commonly used first-line treatments for AGA, but both require long-term use and are associated with limited efficacy and notable side effects [4, 6, 16, 17]. Our study showed that Cur-mEVs effectively remodeled the HF microenvironment and demonstrated significant therapeutic effects in the AGA mouse model, along with excellent safety profiles. Given their natural origin, scalability, and low cost, Cur-mEVs offer new insights for future clinical practices. However, there are some limitations in this study. Due to the inherent hydrophobicity of curcumin and its limited solubility in aqueous systems, the drug loading efficiency (DLE) achieved in our study using electroporation was only 2.2%. Alternative loading strategies, such as ultrasonication or co-incubation, which may potentially enhance the DLE of curcumin, have not yet been explored in this study. In addition, the Cur-mEV group demonstrated a more pronounced therapeutic effect in the AGA model compared to the Cur group. While this improvement may be attributed to the synergistic effect between curcumin and mEVs, whether mEVs enhance the bioavailability of curcumin and thereby improve the efficacy remains unclear in this study. These issues will be key directions for future investigation. In addition, the HF cycle is regulated by multiple pathways and regulatory molecules, which interact with each other to influence HF growth [80, 81]. In this study, we have only investigated the changes in the Wnt/β-catenin signaling pathway and TGF-β1 regulatory molecule, both of which are known to play pivotal roles in the control of the HF cycle [82, 83]. Whether other signaling pathways or regulatory molecules contribute to the regulation of the HF cycle in this study remains to be determined. Furthermore, the mEVs contain a variety of bioactive molecules, and the specific molecules responsible for the observed effects are yet to be identified. Our proteomic analysis of mEVs revealed the presence of multiple cytokines and cytokine receptors, including IGF2, VEGFA, IGF1R and EGFR (Supplementary Table S2). Notably, these proteins are concurrently annotated in the PI3K-Akt, MAPK, and Ras signaling pathways (Supplementary Figure S9). The activation of these pathways has been previously reported to be closely associated with hair regeneration. The interaction between these cytokines and their corresponding receptors plays a pivotal role in triggering the downstream signaling cascades within these pathways [84–88]. Taken together, these findings suggest that various protein components within mEVs contribute to the activation of signaling pathways that promote hair growth. Several studies have utilized EVs sequencing to identify highly enriched miRNAs, followed by validation of their biological functions, with the goal of uncovering potential new therapeutic targets for AGA [66, 89]. These areas will be the focus of our future investigations.

## Conclusion

In this study, the successful preparation of Cur-mEVs was confirmed through various physicochemical characterizations. High concentration of topical application of testosterone demonstrated the most pronounced effect in inducing AGA in the mouse model. In the established AGA mouse model, Cur-mEVs significantly activated HFSCs by synergistically upregulating the Wnt/β-catenin signaling pathway, downregulating TGF-β1 expression, and alleviating inflammation in the HF microenvironment. These combined effects facilitated the transition of the HF cycle and eventually enhanced hair regeneration with excellent biosafety profiles. Overall, our findings demonstrated a promising translational practice and offered new insights for the treatment of AGA through Cur-mEVs.

## Supplementary Material

rbaf051_Supplementary_Data

## Data Availability

Data will be made available on request.
