# Supplementary material for: Curcumin-primed milk-derived extracellular vesicles remodel hair follicle microenvironment for the treatment of androgenetic alopecia
Source: Regen Biomater. 2025 May 30;12:rbaf051. doi: 10.1093/rb/rbaf051 (PMC12306444; doi:10.1093/rb/rbaf051)
Supplement: rbaf051_Supplementary_Data [file rbaf051_supplementary_data.zip › Supporting Information.docx]

**Supporting Information**

**Curcumin-primed milk-derived extracellular vesicles remodel hair follicle microenvironment for the treatment of androgenetic alopecia**

*Chongchao Hou^1,2,#^, Sihua Wang^1,2,#^, Zihang Li^1,2,#^, Qing Huang^2,3^, Yang Jiang^2,4^, Xin Zhou^1,2^, Rongying Ou^5^, Danyang Li^2,^*, Yunsheng Xu^1,2,^**

^1^ Department of Dermatovenereology, The Seventh Affiliated Hospital, Sun Yat-sen University, Shenzhen, 518107, China

^2^ Research Center, The Seventh Affiliated Hospital, Sun Yat-sen University, Shenzhen, 518107, China

^3^ Department of Geriatrics, The Seventh Affiliated Hospital, Sun Yat-sen University, Shenzhen, 518107, China

^4^ Department of Interventional Oncology, The First Affiliated Hospital, Sun Yat-sen University, Guangzhou, 510080, China

^5^ Department of Gynaecology and Obstetrics, The First Affiliated Hospital, Wenzhou Medical University, Wenzhou 325000, China.

^#^ These authors contribute equally.

*****Corresponding authors:**

Dr Danyang Li: lidy55@mail.sysu.edu.cn

Prof Yunsheng Xu: xuysh9@mail.sysu.edu.cn


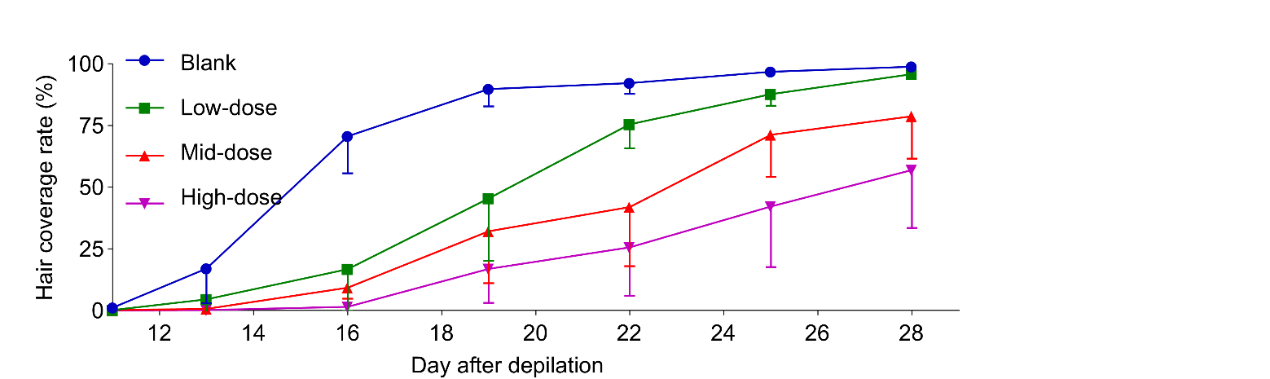


**Figure S1.** Hair coverage rate at different time points after depilation.


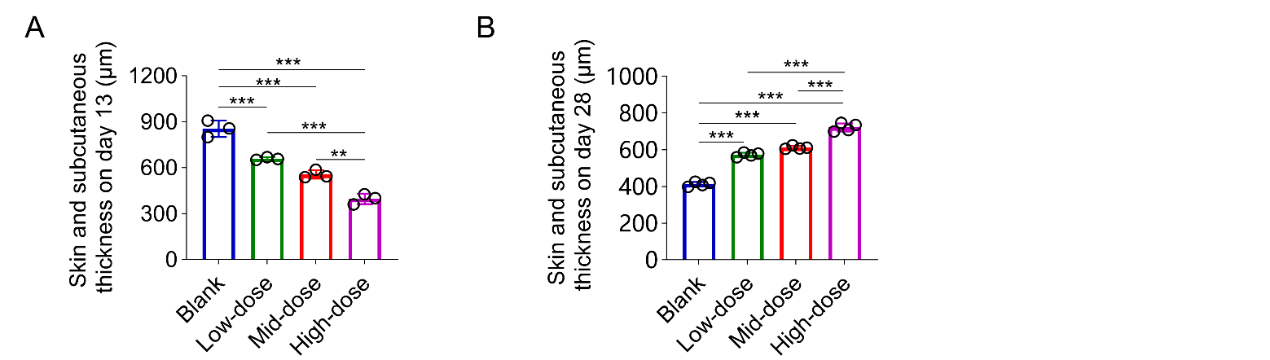


**Figure S2.** Establishment of AGA mouse model with topical application of testosterone. **(A)** Skin and subcutaneous thickness of the skin on day 13 post-depilation. n=3. **(B)** Skin and subcutaneous thickness on day 28 post-depilation. n=4. All results are presented as the mean ± SD. ns, nonsignificant (P > 0.05), *P < 0.05, **P < 0.01, ***P < 0.001.


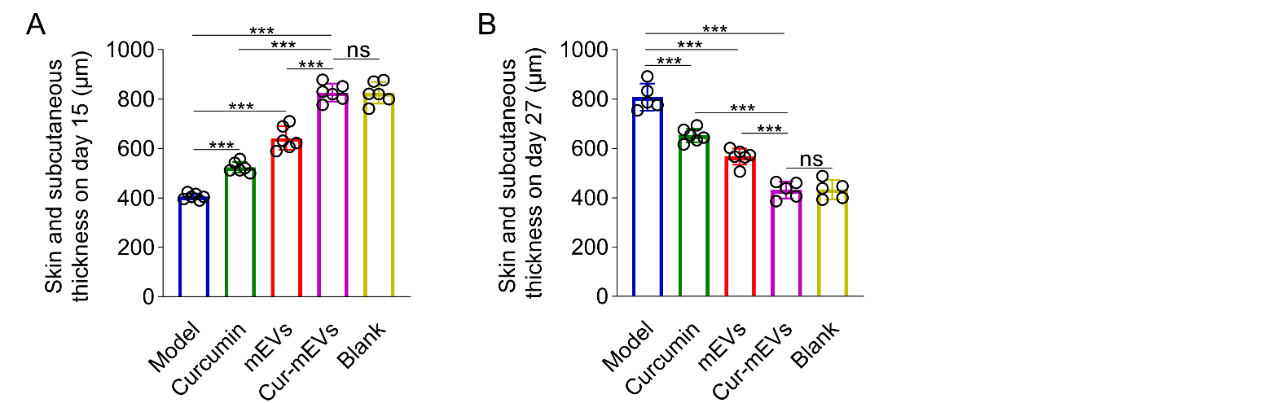


**Figure S3.** Cur-mEVs accelerates the transition of the hair follicle cycle. **(A)** Skin and subcutaneous thickness on day 15 post-depilation. n=6. **(B)** Skin and subcutaneous thickness on day 27 post-depilation. n=5-6. All results are presented as the mean ± SD. ns, nonsignificant (P > 0.05), *P < 0.05, **P < 0.01, ***P < 0.001.


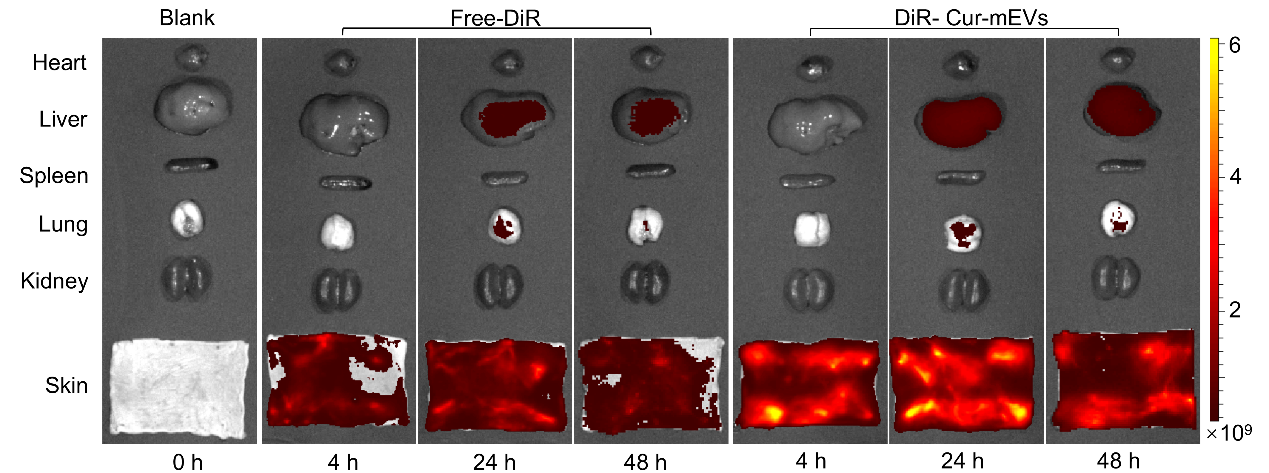


**Figure S4.** After subcutaneous injection, the biodistribution of Free-DiR and DiR-labelled Cur-mEVs in the heart, liver, spleen, lung, kidney and skin *via* IVIS. n=3.


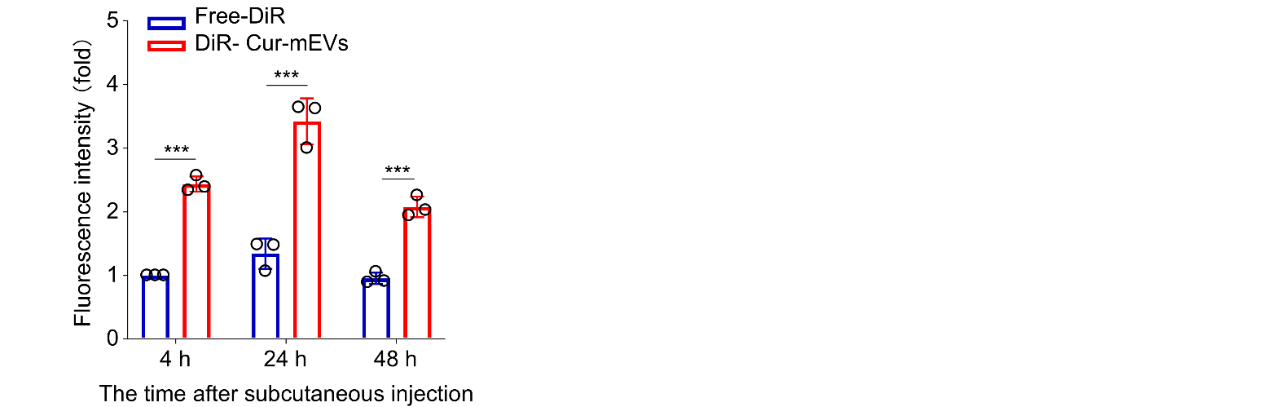


**Figure S5.** The fluorescence intensity of Free-DiR and DiR-labelled Cur-mEVs of the skin. n=3. All results are presented as the mean ± SD. ns, nonsignificant (P > 0.05), *P < 0.05, **P < 0.01, ***P < 0.001.


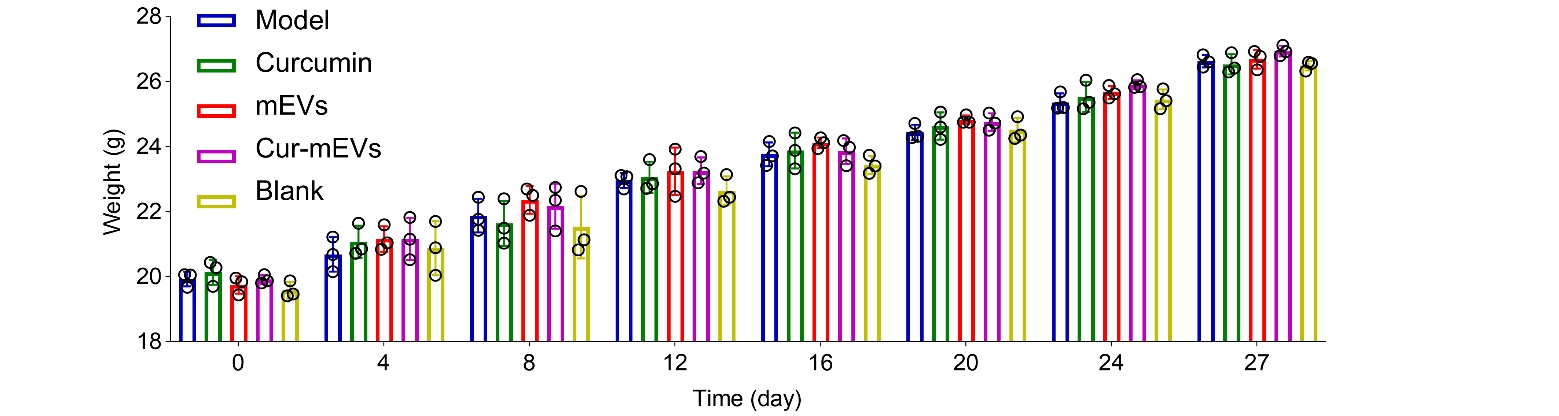


**Figure S6.** The body weight of the mice after therapy. n=3. All results are presented as the mean ± SD. ns, nonsignificant (P > 0.05), *P < 0.05, **P < 0.01, ***P < 0.001.


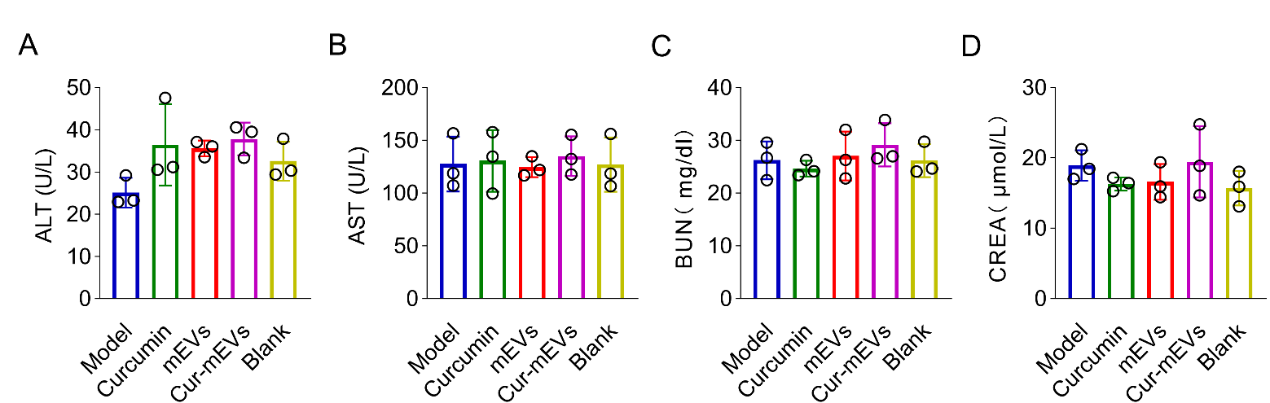


**Figure S7. (A)** and **(B)** Liver function (ALT and AST), **(C)** and **(D)** kidney function (BUN and CREA) were evaluated in different groups after 27 days of treatment to the AGA mouse by blood biochemical examination. n=3. All results are presented as the mean ± SD. ns, nonsignificant (P > 0.05), *P < 0.05, **P < 0.01, ***P < 0.001.


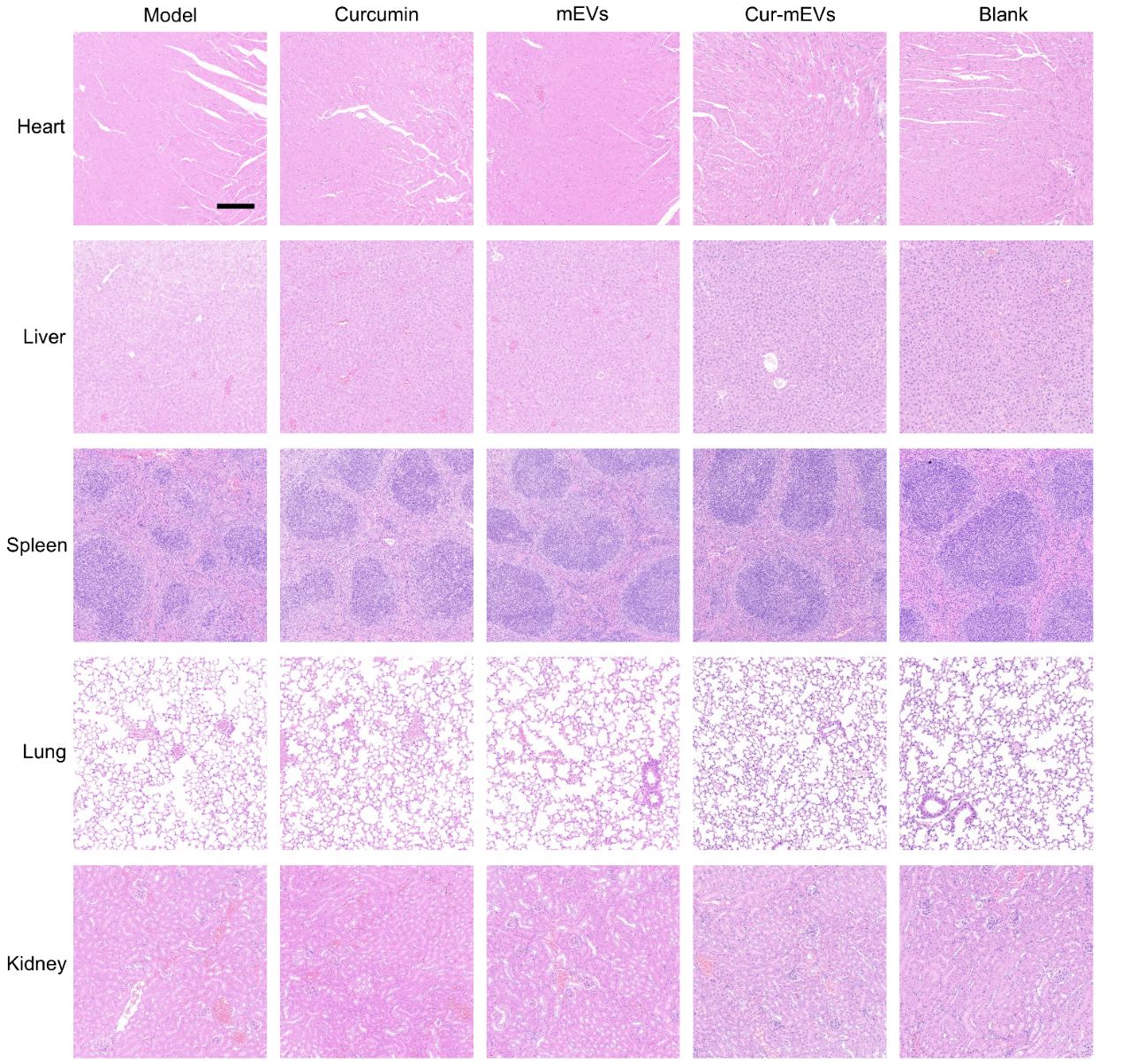


**Figure S8.** H&E staining of the heart, liver, spleen, lung, and kidney in different groups after 27 days of treatment to the AGA mouse, scale bar = 200 µm.

**Table S1.** Hair follicle cycle scoring table

| Hair follicle phase | Sub-phase | Histological Features | Score |
| --- | --- | --- | --- |
| Telogen | - | Miniaturized follicles at sebaceous | 0/400 |
| Early Anagen | I-IIIa | Moderately dense hair bulbs at dermal-subcutaneous junction | 100 |
| Mid Anagen | IIIb-IIIc | Enlarged bulbs in subcutaneous tissue | 200 |
| Late Anagen | IV-VI | Deep subcutaneous bulbs, hair shafts emerging from the skin surface | 300/100 |
| Early Catagen | I-IV | Dense, enlarged hair bulbs at deep subcutaneous | 100 |
| Mid Catagen | V-VI | Moderately shrunken bulbs situated in the superficial subcutaneous | 200 |
| Late Catagen | VII-VIII | Shrinking bulbs at the dermal-subcutaneous junction | 300 |

**Table S2.** KEGG annotated proteins

| **Accession** | **Gene** | **K** | **K_name** |
| --- | --- | --- | --- |
| A0A3Q1LMK4 | CSF1 | K05453 | macrophage colony-stimulating factor 1 |
| A0A3Q1LMP2 | KRAS | K07827 | GTPase Kras |
| A0A3Q1LPW3 | EPHA2 | K05103 | Eph receptor A2 |
| A0A3Q1M7B5 | MAPK3 | K04371 | mitogen-activated protein kinase 1/3 |
| A0A3Q1MHB0 | EGFR | K04361 | epidermal growth factor receptor |
| A0A3Q1MQ45 | AKT2 | K04456 | RAC serine/threonine-protein kinase |
| A0AAA9SNF6 | MAPK3 | K04371 | mitogen-activated protein kinase 1/3 |
| A0AAA9T9H0 | NRAS | K07828 | GTPase NRas |
| A0AAA9TR33 | PDGFC | K05450 | platelet derived growth factor C/D |
| A0AAA9U2K8 | MAP2K2 | K04369 | mitogen-activated protein kinase kinase 2 |
| A7E3S4 | RAF1 | K04366 | RAF proto-oncogene serine/threonine-protein kinase |
| F6RGB4 | GRB2 | K04364 | growth factor receptor-bound protein 2 |
| G3N1U2 | HRAS | K02833 | GTPase HRas |
| P04409 | PRKCA | K02677 | classical protein kinase C alpha type |
| P07456 | IGF2 | K13769 | insulin-like growth factor 2 |
| P15691 | VEGFA | K05448 | vascular endothelial growth factor A |
| P46196 | MAPK1 | K04371 | mitogen-activated protein kinase 1/3 |
| P62998 | RAC1 | K04392 | Ras-related C3 botulinum toxin substrate 1 |
| Q05688 | IGF1R | K05087 | insulin-like growth factor 1 receptor |
| Q0VD16 | MAP2K1 | K04368 | mitogen-activated protein kinase kinase 1 |
| Q28024 | GNG12 | K04347 | guanine nucleotide-binding protein G(I)/G(S)/G(O) subunit gamma-12 |
| Q3ZC64 | EFNA1 | K05462 | ephrin-A |
| Q769I5 | MET | K05099 | proto-oncogene tyrosine-protein kinase Met |
| Q8MJ50 | OSTF1 | K04364 | growth factor receptor-bound protein 2 |

**Annotation:** The **Accession** represents the protein's ID or identifier, **Gene** indicates the gene name of the protein, **K** corresponds to the sequence ID in the KEGG database for protein alignment, and **K_name** provides the description of the KEGG sequence ID.


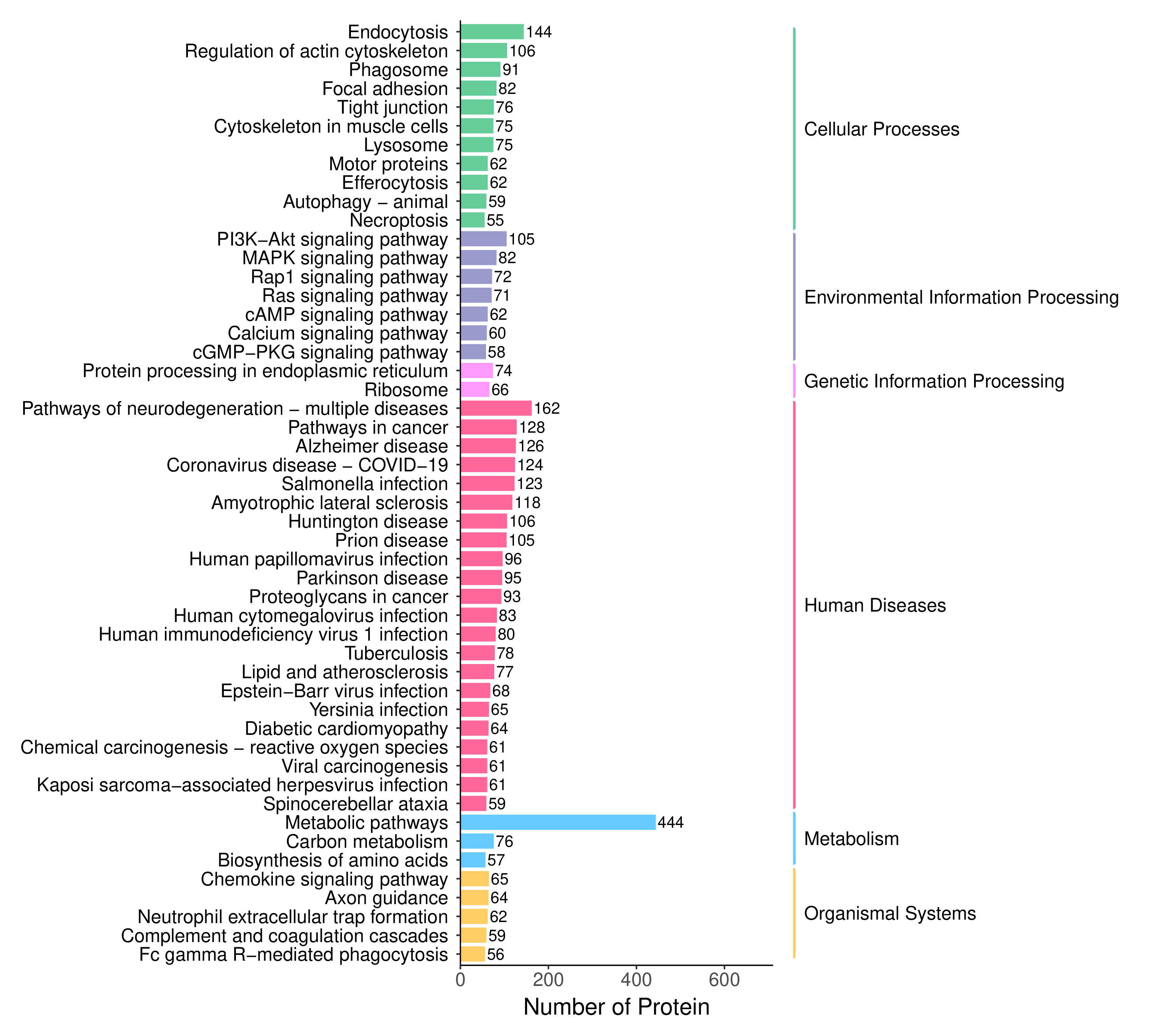


**Figure S9.** Bar plot of KEGG annotation results for proteins.
